# Elective Cardiopulmonary Bypass (CPB) Surgery After COVID-19: Vasoactive Needs and Early Complications—A Prospective Study

**DOI:** 10.3390/jcm14238290

**Published:** 2025-11-21

**Authors:** Cornelia-Elena Predoi, Daniela Carmen Filipescu, Mihai Gabriel Stefan, Niculae Iordache

**Affiliations:** 1 Emergency Institute of Cardiovascular Disease “Prof. Dr. CC Iliescu”, 022322 Bucharest, Romania; corneliaelenapredoi@gmail.com (C.-E.P.); 1danielaf@gmail.com (D.C.F.); niordache@gmail.com (N.I.); 2 Faculty of Medicine, University of Medicine and Pharmacy “Carol Davila”, 020021 Bucharest, Romania; 3 Sf. Ioan Clinical Emergency Hospital, 042122 Bucharest, Romania

**Keywords:** COVID-19, cardiopulmonary bypass, vasopressors, perioperative, cardiac surgery

## Abstract

**Background/Objectives**: Whether a remote history of SARS-CoV-2 infection independently affects early haemodynamic stability after elective cardiopulmonary bypass (CPB) remains uncertain. We evaluated whether prior COVID-19 (>7 weeks before surgery) was associated with postoperative vasopressor requirements or early complications in adults undergoing elective CPB. **Methods**: We conducted a single-centre prospective cohort study including adults (≥18 years) scheduled for elective on-pump coronary, valve, or combined cardiac surgery between 1 August 2022 and 30 October 2023. Patients undergoing emergency procedures or surgery < 7 weeks after infection were excluded. The exposure was a documented history of COVID-19 for >7 weeks preoperatively. The primary outcome was postoperative vasopressor use within 24 h of ICU admission; secondary outcomes included inotrope use, arrhythmias, acute cardiac or respiratory failure, pneumonia, acute kidney injury (KDIGO), delirium, stroke, length of stay, and mortality. Multivariable logistic regression adjusted for age, CPB duration, obesity, anaemia, chronic kidney disease, sex, EuroSCORE I, left ventricular ejection fraction, and procedure type. **Results:** Of 351 screened patients, 280 elective CPB cases were analyzed; 101 (36.1%) had prior COVID-19. Vasopressor use occurred in 151/280 (53.9%) patients, with no difference between COVID and non-COVID groups (53.5% vs. 54.2%; *p* = 1.00). Prior COVID-19 was not associated with vasopressor requirement (adjusted OR 0.94, 95% CI 0.56–1.59). Independent predictors were longer CPB duration (aOR 2.80 per hour; *p* < 0.001) and older age (aOR 1.028 per year; *p* = 0.02). Secondary outcomes, including organ dysfunction and mortality, did not differ between groups. **Conclusions:** In adults undergoing elective CPB ≥ 7 weeks after SARS-CoV-2 infection, prior COVID-19 did not increase early vasopressor needs or short-term postoperative complications. Haemodynamic requirements were primarily driven by CPB duration and age. Further research using dose-standardized vasoactive metrics and formal COVID-19 severity stratification is warranted.

## 1. Introduction

Early in the pandemic, observational cohorts reported increased perioperative morbidity and mortality when surgery was performed soon after SARS-CoV-2 infection. The international COVIDSurg/GlobalSurg study showed that the excess 30-day postoperative mortality persisted up to six weeks from diagnosis and approached baseline from seven weeks onward, informing the widely adopted recommendation to defer elective procedures for ≥7 weeks following infection [1]. Subsequent analyses refined this signal, indicating that risk depends on timing, illness severity, and perioperative context. For example, in a large U.S. cohort, active COVID-19 at the time of surgery was associated with more cardiac complications, whereas resolved infection (>4 weeks) was not, compared with COVID-negative controls [2]. In cancer surgery, patients recovering from mild-to-moderate COVID-19 who underwent elective procedures after a short convalescence showed no increase in composite adverse events versus matched controls [3].

Synthesizing the heterogeneous literature, a systematic review found that for patients operated ≥7 weeks after mostly asymptomatic or mild infection, postoperative event rates were low and comparable to non-COVID cohorts: pneumonia ~1.66%, pulmonary embolism ~1.47%, arrhythmias ~2.57%, myocardial injury ~1.06%, venous thromboembolism (VTE) ~2.78%, and an overall mortality of 2.27% (607/26,688). Elevated risk appears concentrated in subgroups with moderate–severe antecedent disease or recent hospitalization, and in selected complex procedures [4]. Conversely, in a large administrative analysis of commercially insured adults, prior resolved COVID-19 was not independently associated with 90-day postoperative venous thromboembolism (VTE) after adjustment; surgical exposure itself remained the dominant driver of thrombotic risk [5].

Cardiopulmonary bypass (CPB) creates a unique perioperative physiological environment that limits the applicability of COVID-related risk estimates derived from non-cardiac surgery. CPB exposes blood to non-endothelial surfaces and non-physiological flow, triggering a robust systemic inflammatory response, complement activation, nitric oxide–mediated vasodilation, and endothelial injury—mechanisms that contribute to postoperative vasoplegia and altered hemodynamic responsiveness [6,7]. These perturbations differ fundamentally from those encountered in non-cardiac procedures, where hemodynamic instability is typically less dominated by extracorporeal circulation–related inflammatory pathways. Because SARS-CoV-2 infection itself has been associated with chronic endothelial dysfunction and impaired vascular reactivity [8], it is biologically plausible that residual post-COVID microvascular alterations could interact with CPB-induced endothelial stress. This distinct mechanistic interface underscores the need to evaluate postoperative vasoactive requirements specifically in patients undergoing CPB, rather than extrapolating from heterogeneous perioperative data in non-cardiac surgical populations.

Whether a history of COVID-19 continues to influence early postoperative haemodynamics and organ dysfunction after elective CPB—a setting characterized by systemic inflammation, endothelial activation, and fluid/vasoactive shifts—remains less certain, as most prior studies focused on non-cardiac surgery or mixed specialties [1,2,3,5]. Clarifying this association is clinically relevant for perioperative planning in cardiac anaesthesia and intensive care, particularly regarding vasoactive requirements, respiratory and renal complications, length of stay, and short-term mortality.

Accordingly, the present study compares postoperative outcomes in adults undergoing elective CPB surgery with and without a history of COVID-19 (>7 weeks preoperatively), with a primary focus on vasoactive support (vasopressors/inotropes) and secondary evaluation of cardiopulmonary, neurological, and renal complications, ICU/hospital length of stay, and mortality [1,2,3,5].

## 2. Materials and Methods

The study was a single-centre prospective cohort of consecutive adults undergoing elective cardiac surgery with CPB conducted at the Emergency Institute for Cardiovascular Disease ‘Professor Doctor C.C. Iliescu’, Bucharest, Romania, between 1 August 2022 and 30 October 2023. This study is registered at ClinicalTrials.gov (NCT05752162) and was approved by the institutional ethics committee (Approval No. 20083/11 July 2022).

The inclusion criteria for this study were: adults (≥18 years) undergoing elective on-pump cardiac surgery (coronary artery bypass grafting—CABG, valve surgery, or combined procedures), while the exclusion criteria were: emergency/urgent procedures; off-pump surgery; surgery performed <7 weeks after confirmed SARS-CoV-2 infection; and cases with missing primary outcome data.

A history of COVID-19 was defined as documented SARS-CoV-2 infection > 7 weeks prior to surgery, confirmed by laboratory records and/or medical documentation, in line with contemporary perioperative guidance. For patients with prior COVID-19, we recorded, where available, whether the infection had been managed in the outpatient setting or had required hospital admission for COVID-19 pneumonia, and the calendar year of infection (2020–2023) to approximate variant/vaccine eras. Because all patients with prior infection in this cohort met the ≥7-week criterion by design and detailed gradation of clinical severity (e.g., asymptomatic vs. mild vs. moderate–severe) was not consistently documented in the health record, we did not further stratify analyses by severity class or by finer time intervals beyond this threshold. The comparator comprised patients without known prior COVID-19. Where available, we recorded vaccination status (≥1 dose vs. none) and calendar year of infection (2020–2023) to contextualize variant/vaccine eras. Additional pre-specified sensitivity analyses explored the potential impact of vaccination and infection era. These included (i) restricting the cohort to patients who had received ≥1 COVID-19 vaccine dose before surgery and (ii) incorporating calendar year of infection (2020–2023) as an additional covariate among patients with prior COVID-19.

The primary outcome was postoperative vasopressor use, defined as any continuous infusion of norepinephrine, epinephrine, vasopressin, or equivalent maintained within the first 24 h of ICU admission.

The secondary outcomes were inotrope use, arrhythmias, acute cardiac failure (low-output state requiring inotrope or mechanical support), acute respiratory failure, pneumonia, AKI, delirium, stroke (new focal deficit > 24 h with imaging confirmation), ICU and hospital LOS, and in-hospital/30-day mortality.

All secondary outcomes were defined using standardized and validated clinical criteria. Acute kidney injury was classified according to the KDIGO serum-creatinine criteria; pneumonia was defined using CDC/NHSN criteria; and delirium was assessed with the CAM-ICU instrument. Acute respiratory failure required re-intubation or non-invasive ventilation with a PaO_2_/FiO_2_ ratio < 300 mmHg. Arrhythmias were defined as new-onset atrial fibrillation/flutter or sustained ventricular arrhythmias requiring treatment.

Although infusion start/stop times and vasoactive dosing were captured inconsistently in the electronic health record, preventing calculation of dose-standardized scores such as the Vasoactive-Inotropic Score (VIS), any continuous infusion of norepinephrine, epinephrine, vasopressin, dobutamine, or milrinone within the first 24 h was recorded as vasoactive support. The limitations related to dose-standardization are addressed in the Discussion

Pre-specified covariates included age, sex, body mass index (BMI) (with obesity defined as BMI > 30 kg/m^2^), ASA class, NYHA class, EuroSCORE I (institutionally recorded, while EuroSCORE II was not available for all patients), smoking status, preoperative anaemia (Hb < 12 g/dL for women; <13 g/dL for men), chronic kidney disease (CKD) (eGFR < 60 mL/min/1.73 m^2^), diabetes, serum creatinine, CPB duration and aortic cross-clamp time, procedure type (CABG/valve/combined/other), left ventricular ejection fraction (LVEF) categorized as ≥50%, 40–49%, or <40% and calendar year of surgery (2022 vs. 2023). Stable provider identifiers were unavailable, and software limitations precluded the use of clustered standard errors.

Data were abstracted from the health record using a standardized extraction form with a priori variable definitions by two independent investigators; discrepancies were resolved by consensus. Procedures for variable definition, capture, and quality control followed established good-practice principles for protocol standardization and reproducible analytical workflows.

The analytic cohort comprised 280 elective CPB cases; 71 emergency procedures were excluded. With 151 vasopressor events, the multivariable model satisfied the events-per-variable ≥ 10 heuristic. The primary analysis used complete cases.

Continuous variables are reported as mean ± SD when approximately normally distributed and as median (interquartile range- IQR) when skewed or ordinal (e.g., ICU LOS, hospital LOS, ventilation hours, ASA, NYHA). Between-group comparisons employed independent-samples *t*-tests or Mann–Whitney U tests, as appropriate; categorical variables were compared using χ^2^ or Fisher’s exact tests.

For the primary endpoint, we fitted a binary logistic regression model. Univariable models estimated crude odds ratios (ORs) with 95% CIs. The multivariable model included history of COVID-19, age, CPB duration, obesity (BMI > 30), anaemia, CKD, and clinically relevant covariates (sex, EuroSCORE I, LVEF category, procedure type). Linearity in the logit for continuous predictors was assessed (Box–Tidwell); for interpretability, age effects are additionally presented per decade. Multicollinearity was evaluated using variance inflation factors (VIF < 5). Model performance was assessed using the Hosmer–Lemeshow goodness-of-fit test, area under the ROC curve (AUC), and calibration plots. Two-sided α = 0.05 was used for statistical significance.

Pre-specified sensitivity analyses included: (i) excluding infections from 2020; (ii) restricting the cohort to vaccinated patients; and (iii) stratifying by procedure type (CABG vs. valve vs. combined). An interaction between history of COVID-19 and age was explored a priori.

Analyses were performed in IBM SPSS Statistics, version 26 (IBM Corp., Armonk, NY, USA).

To enhance reproducibility, variable definitions, data capture and quality control procedures were standardized a priori and mirrored good-practice principles from methodological optimization studies [9].

### Use of Artificial Intelligence Tools

Artificial intelligence–based tools were used to assist with language editing of this manuscript

## 3. Results

During the study period, 351 cardiac procedures with CPB were screened. After exclusion of 71 emergency or urgent cases, the final analytic cohort comprised 280 elective CPB surgeries. Procedure types were coronary artery bypass grafting (CABG) in 82/280 (29.3%), isolated aortic valve replacement in 68/280 (24.3%), isolated mitral valve replacement in 24/280 (8.6%), combined/complex procedures in 78/280 (27.9%), and other procedures in 28/280 (10.0%). Preoperative LV function was preserved (LVEF ≥ 50%) in 235/280 (83.9%), mildly reduced (40–49%) in 31/280 (11.1%), and reduced (<40%) in 14/280 (5.0%).

Of the 280 patients, 101 (36.1%) had a documented history of COVID-19 > 7 weeks before surgery and 179 (63.9%) had no known prior infection. The COVID-19 group therefore reflects patients with remote infection (>7 weeks) rather than those with recent or active disease; detailed severity grading was incompletely available and is described in the Limitations.

### 3.1. Baseline Characteristics

Baseline characteristics are summarized in Table 1. Median age was similar between patients with and without prior COVID-19 (66 [IQR 54–69] vs. 64 [55–69] years; *p* = 0.939). A higher proportion of men was observed in the no-COVID group (69.8% vs. 57.4%; *p* = 0.049), and sex was therefore included as an adjustment covariate in the multivariable model.

Other preoperative characteristics were broadly comparable between groups. Median ASA class was 3 [3,4] in both groups (*p* = 0.284), and median NYHA class was 3 [2,3] in both groups (*p* = 0.072). EuroSCORE I was similar (5 [3–6] vs. 4 [3–6]; *p* = 0.269), as were median preoperative creatinine (0.96 [0.74–1.16] vs. 0.95 [0.82–1.14] mg/dL; *p* = 0.413), CPB duration (1.55 [1.33–1.90] vs. 1.56 [1.26–1.98] h; *p* = 0.946), and aortic cross-clamp time (1.08 [0.88–1.36] vs. 1.10 [0.81–1.43] h; *p* = 0.886).

Mean preoperative BMI was also similar (27.8 ± 4.9 vs. 27.9 ± 5.0 kg/m^2^; *p* = 0.752). Trends toward more preoperative anaemia in the COVID-19 group (38.6% vs. 27.4%; *p* = 0.061) did not reach conventional statistical significance and were interpreted cautiously. CKD, diabetes, smoking status, and vaccination status (≥1 COVID-19 vaccine dose before surgery in 63.4% vs. 60.8%; *p* = 0.703) were comparable across groups (all *p* > 0.45).

### 3.2. Primary Outcome: Postoperative Vasopressor Use

Overall, 151/280 patients (53.9%) required vasopressor support within the first 24 h after ICU admission. Rates were virtually identical in patients with and without prior COVID-19 (54/101 [53.5%] vs. 97/179 [54.2%]; *p* = 1.000) (Table 2).

In univariable logistic regression, longer CPB duration and older age were associated with higher odds of vasopressor use, whereas obesity (BMI > 30 kg/m^2^) was associated with lower odds (Table 3). Prior COVID-19, anaemia, and CKD were not significantly associated with vasopressor requirement.

**Figure 1:**
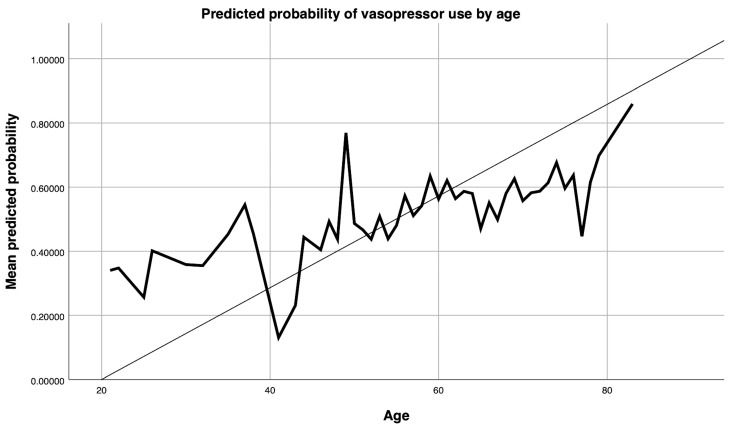
Predicted probability of vasopressor use by age. Predicted probabilities in Figure 1 were obtained from the multivariable logistic regression model adjusted for age, CPB duration, obesity (BMI > 30 kg/m^2^), preoperative anemia, chronic kidney disease, sex, EuroSCORE I. The *x*-axis represents age in years (range 20–85), and the *y*-axis represents the mean predicted probability of vasopressor use (0.00–1.00). The solid line represents the mean predicted probability of vasopressor use, and the dashed lines represent the 95% confidence interval.

In the multivariable model adjusting for history of COVID-19, age, CPB duration, obesity, anaemia, CKD, sex, EuroSCORE I, LVEF category, and procedure type, two variables remained independently associated with vasopressor use:

- CPB duration (per hour): adjusted OR (2.80, 95% CI 1.72–4.54; *p* < 0.001).
- Age (per year): adjusted OR (1.028, 95% CI 1.004–1.052; *p* = 0.020), equivalent to ~32% higher odds per decade (Figure 1).

By contrast, the history of COVID-19 showed no independent association with vasopressor requirement (adjusted OR 0.94, 95% CI 0.56–1.59; *p* = 0.812). Anaemia (adjusted OR 1.19, 95% CI 0.68–2.08; *p* = 0.535) and CKD (adjusted OR 1.17, 95% CI 0.57–2.42; *p* = 0.672) were likewise not significant. Obesity remained inversely associated with vasopressor use (adjusted OR 0.50, 95% CI 0.29–0.86; *p* = 0.012); this counterintuitive finding may reflect case-mix or residual confounding and should be interpreted with caution.

**Figure 2:**
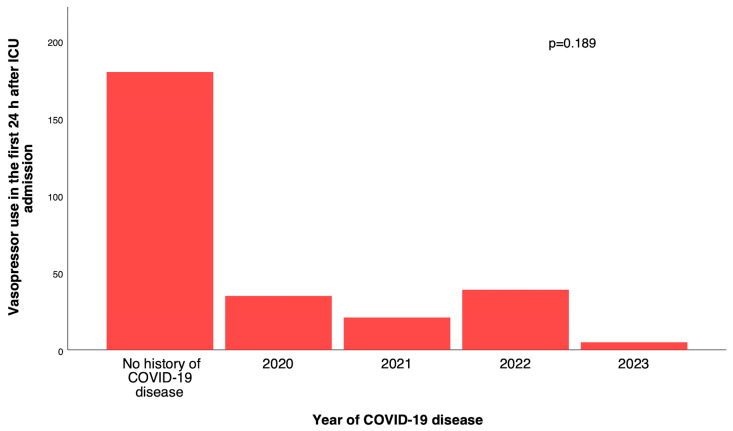
Postoperative vasopressor use by year of prior COVID-19 diagnosis.

A descriptive analysis stratifying vasopressor use by the calendar year of prior COVID-19 infection (none, 2020–2023) showed no significant association between year of infection and vasopressor requirement (*p* = 0.189, χ^2^), although numbers within year-specific strata were small (Figure 2). Calendar year of surgery (2023 vs. 2022) was also not associated with vasopressor use (adjusted OR 0.68, 95% CI 0.39–1.20; *p* = 0.18), and including this variable did not materially alter the estimates for history of COVID-19, CPB duration, or age (all adjusted OR shifts ≤ 10%).

Figure 2 illustrates the distribution of postoperative vasopressor use according to the year of prior COVID-19 infection. Patients with no history of COVID-19 comprised the largest group requiring vasopressors (n = 97). Among patients with previous COVID-19, vasopressor use appeared similar across infection years 2020–2023, with no statistically significant difference between groups (χ^2^ *p* = 0.189). These findings indicate that the calendar year of prior SARS-CoV-2 infection—representing different pandemic waves and variants—did not materially influence postoperative vasopressor requirement.

### 3.3. Secondary Outcomes

Secondary postoperative outcomes are summarized in Table 2. There were no statistically significant differences between patients with and without prior COVID-19 in inotrope use, cardiopulmonary complications, renal or neurological outcomes, length of stay, or mortality.

Inotrope use within 24 h occurred in 81/280 (28.9%) patients overall: 31/101 (30.7%) with prior COVID-19 and 50/179 (27.9%) without (*p* = 0.681). Arrhythmias were recorded in 65/280 (23.2%) patients (24.8% vs. 22.3%; *p* = 0.661). Rates of acute cardiac failure (10.9% vs. 10.6%; *p* = 1.000), acute respiratory failure (19.8% vs. 25.1%; *p* = 0.377), pneumonia (3.0% vs. 3.4%; *p* = 1.000), and AKI (7.9% vs. 11.7%; *p* = 0.415) were low and similar across groups.

Median duration of invasive ventilation was 7 [5–10] hours in the overall cohort (8 [5–12] vs. 7 [5–10] hours in patients with and without prior COVID-19; *p* = 0.351). Median ICU length of stay was 3 [2–3] days in both groups (*p* = 0.919), and median hospital stay was 8 [7–10] days overall (8 [7–9] vs. 8 [7–10] days; *p* = 0.132).

Neurological events were infrequent: delirium occurred in 18/280 (6.4%) patients (5.9% vs. 6.7%; *p* = 1.000), and stroke occurred in 3/280 (1.1%), all in the no-COVID group (0% vs. 1.7%; *p* = 0.555). In-hospital mortality was low and did not differ significantly between groups (1/101 [1.0%] vs. 4/179 [2.2%]; *p* = 0.657).

Taken together, these results indicate that, in adults undergoing elective CPB at least 7 weeks after SARS-CoV-2 infection, prior COVID-19 was not associated with higher vasopressor requirements or excess early postoperative morbidity or mortality, whereas longer CPB duration and older age emerged as the principal determinants of postoperative vasoactive support.

## 4. Discussion

In this single-centre cohort of 280 elective CPB procedures, a remote history of COVID-19 (>7 weeks) was not associated with increased postoperative vasopressor requirement or other early adverse outcomes. Crude vasopressor use was virtually identical (53.5% with prior COVID-19 vs. 54.2% without), and the adjusted association was null (aOR 0.94; 95% CI 0.56–1.59). Instead, CPB duration and age were the principal determinants of vasoactive support (aOR 2.80 per hour of CPB and aOR 1.028 per year, respectively). Rates of inotrope use, arrhythmias, acute cardiac and respiratory failure, AKI, LOS, and mortality were likewise similar between exposure groups. These results suggest that—once an adequate convalescence interval has elapsed—prior SARS-CoV-2 infection does not independently worsen early haemodynamic stability after elective CPB.

Our results are consistent with perioperative guidance and expert consensus recommending deferral of elective surgery for several weeks after infection and individualized risk assessment thereafter. While most consensus statements emphasize timing windows and overall risk rather than vasoactive endpoints, they collectively support proceeding once the early high-risk period has passed and the patient has recovered clinically—an approach concordant with our CPB-specific data [10].

Outside the operating room, multiple ICU investigations show that vasopressor exposure in COVID-19 tracks with illness severity and worse outcomes. A systematic review and meta-analysis associated vasopressor use with higher mortality and AKI in critically ill COVID-19 patients, acknowledging confounding by indication and substantial heterogeneity [11]. A large multicentre cohort demonstrated a dose–response pattern: mortality rose with the number of vasopressors required [12]. National data from France documented temporal changes in organ-support practices across pandemic waves—including vasopressors—alongside evolving case-mix and outcomes [13]. Multicentre registry data from sub-Saharan Africa provide complementary benchmarks for vasopressor utilization and ICU outcomes in resource-limited settings, underscoring that vasoactive requirements reflect severity and context rather than a single disease label [10]. Against this background, our finding of no excess vasopressor need after remote COVID-19 in elective CPB is clinically reassuring and helps fill an evidence gap in the perioperative domain.

CPB triggers non-physiological flow, a systemic inflammatory response with endothelial activation, and vasoplegia—mechanisms that increase the likelihood of postoperative vasoactive support [6,7]. Residual post-COVID pathophysiology—persistent endothelial and microvascular dysfunction and autonomic dysregulation—has been documented months after infection and could plausibly augment vasodilatory tone and catecholamine resistance during CPB, thereby amplifying perioperative vasoplegia in susceptible patients [8,14]. In our adjusted model, however, procedure-related burden (CPB time) and age outweighed any signal from COVID-19 history, and the inverse association with obesity—though statistically significant—likely reflects case-mix or residual confounding rather than a protective effect and should be interpreted cautiously.

Although neurologic complications were infrequent in our cohort, peri-COVID neurologic syndromes remain relevant to perioperative care. An international prospective study of patients with COVID-19 and acute encephalopathy or coma reported substantial morbidity, reinforcing vigilance for neurologic vulnerability in this population [15]. Contemporary perioperative reviews also highlight the post-acute sequelae of COVID-19 (PASC/long-COVID)—autonomic symptoms, exertional intolerance, and cognitive complaints—that may influence perioperative trajectories even when traditional complications are rare [16,17]. These data support continued screening for delirium, optimization of rehabilitation, and tailored hemodynamic goals in selected patients.

For patients with remote, uncomplicated COVID-19, elective CPB can likely proceed under standard haemodynamic targets without anticipating an excess need for vasopressors solely due to prior infection. Perioperative optimization should continue to prioritize modifiable drivers—limiting CPB duration, meticulous temperature and vasodilator management, correction of haemodilution/hypocalcaemia, and judicious use of norepinephrine/vasopressin for vasoplegia—rather than COVID-19 history per se. These implications are aligned with expert consensus on timing and perioperative management after SARS-CoV-2 infection [10].

Beyond the perioperative literature on timing and noncardiac procedures, several cardiac surgery series help contextualize our results. In a multicentre analysis of hospital-associated (in-hospital) SARS-CoV-2 infections among cardiac surgical patients, Spadaccio et al. reported higher-than-expected postoperative mortality, with the excess risk most pronounced when infection occurred early in the postoperative course—a scenario mechanistically distinct from our cohort of patients with remote infections presenting for elective CPB. These data reinforce that timing relative to surgery and infection setting (community-resolved vs nosocomial/early postoperative) are critical determinants of risk, and they align with our observation that a remote history of COVID-19 was not associated with increased vasoactive requirements or adverse events [18]. Focusing specifically on CABG, two controlled studies provide complementary signals. Şahin et al. (prospective, controlled) found no significant differences in early postoperative mortality or vascular/thromboembolic outcomes in patients with a history of mild COVID-19 who underwent CABG after a ≥4-week recovery interval—suggesting that once convalescence is adequate, early vascular outcomes are not adversely affected. This is directionally consistent with our neutral findings on haemodynamic support after ≥7 weeks [19]. In a single-centre comparative series of off-pump CABG, Gabriyelyan et al. likewise reported perioperative courses without clear excess morbidity in patients with prior COVID-19 versus controls, further supporting the notion that resolved infection per se may not be an independent driver of early postoperative instability [20]. Conversely, Erçen Diken et al. examined patients with documented prior COVID-19 pneumonia undergoing CABG and observed higher rates of pulmonary complications—while mortality remained similar—and no clear modification by vaccination status. Taken together, these data suggest that the severity of antecedent disease (e.g., prior pneumonia) may still mark a subgroup at higher pulmonary risk, even when mortality is unaffected. Importantly, our cohort (predominantly remote, uncomplicated infections) did not show haemodynamic penalties, implying that any residual risk is likely organ-system specific (pulmonary) rather than a generalized propensity to vasoplegia or vasopressor dependence [21]. Cardiac surgery studies to date converge on a coherent message: nosocomial/early postoperative infections are hazardous; remote and mild prior infections after an adequate interval appear safe with respect to early vascular/haemodynamic endpoints; and prior moderate–severe disease (pneumonia) may carry lingering pulmonary vulnerability. These patterns dovetail with our finding that procedure-related factors (e.g., CPB duration) and age, rather than COVID-19 history, are the dominant determinants of vasopressor use after elective CPB.

Regarding the strengths of our study we can say that it is a prospective study and includes prespecified definitions, dual-review data abstraction, and multivariable modelling with adequate events-per-variable.

## 5. Limitations

The single-centre design and the absence of dose-standardized vasoactive metrics are the first limitations to mention. Because the electronic health record did not consistently capture infusion rates or precise start/stop times, norepinephrine-equivalents, the vasoactive-inotrope score (VIS), and 24 h exposure AUC could not be derived. Also, we lack patient-reported outcomes, which are increasingly recognized as important to postoperative recovery. Our analysis was not designed to capture quality-of-life outcomes. Risk adjustment used EuroSCORE I because EuroSCORE II was incompletely captured during the study period, which may introduce modest model misspecification. In addition, stable surgeon/anaesthetist identifiers were unavailable, precluding the use of cluster-robust variance estimators; unmodelled provider-level clustering could therefore lead to underestimated standard errors. Future studies with complete provider identifiers should employ mixed-effects or cluster-robust approaches. Our cohort specifically examined patients with remote SARS-CoV-2 infection (all >7 weeks preoperatively), consistent with prevailing perioperative recommendations. While we captured calendar year of infection and, where documented, whether COVID-19 required hospital admission, systematic grading of preoperative disease severity (e.g., asymptomatic, mild, moderate–severe) and precise time-since-infection beyond the ≥7-week threshold was incomplete and precluded robust subgroup analyses. As such, our findings are most applicable to patients with remote, predominantly non-critical infection, and may not fully generalize to those with recent or severe COVID-19. Although we collected vaccination status and infection era and incorporated them in sensitivity analyses, residual confounding related to evolving variants, vaccination schedules, and treatment strategies across pandemic waves cannot be fully excluded.

Nevertheless, consistent with the broader surgical literature future cardiac perioperative studies should integrate patient-reported outcomes to provide a more comprehensive appraisal of recovery beyond short-term complications and length of stay [22].

Future work should: (i) adopt standardized vasoplegia phenotyping (lactate, arterial elastance, vasoactive-inotrope scores) to enable cross-study comparability; (ii) investigate agent choice and dose–response in CPB vasoplegia; (iii) integrate PASC/long-COVID screening into pre-assessment; and (iv) evaluate longer-term functional and quality-of-life outcomes beyond the index hospitalization [16,17].

## 6. Conclusions

In this prospective cohort of 280 adults undergoing elective cardiopulmonary bypass, a remote history of SARS-CoV-2 infection (>7 weeks preoperatively) was not associated with increased postoperative vasopressor requirements or excess early morbidity. Vasopressor use was almost identical in patients with and without prior COVID-19, and the adjusted analysis confirmed the absence of an independent association. Instead, CPB duration and older age emerged as the primary determinants of postoperative vasoactive support. Secondary outcomes—including cardiopulmonary, renal, and neurological complications, length of stay, and mortality—were likewise comparable between groups.

These findings suggest that once a sufficient convalescence interval has elapsed, prior COVID-19 infection alone should not warrant altered hemodynamic targets or anticipatory escalation of vasoactive therapy during elective CPB. Standard perioperative management remains appropriate, with continued focus on modifiable intraoperative factors such as CPB time.

Future studies should incorporate standardized vasoplegia phenotyping, dose-standardized vasoactive metrics, robust COVID-19 severity classification, and longer-term functional outcomes to better delineate whether specific subgroups (e.g., patients with prior moderate–severe COVID-19) may benefit from tailored perioperative strategies.

## Figures and Tables

**Table 1:** Baseline characteristics.

Patients’ Characteristics	All Cohort—280 Patients	History of COVID-19—101 Patients	No History of COVID-19—179 Patients	*p*-Value
Age (years)—median [IQR]	65 [55–69]	66 [54–69]	64 [55–69]	0.939
Male gender (n, %)	183 (65.4)	58 (57.4)	125 (69.8)	0.049
ASA—median [IQR]	3 [3–4]	3 [3–4]	3 [3–5]	0.284
NYHA—median [IQR]	3 [2–3]	3 [2–3]	3 [2–3]	0.072
EuroSCORE I—median [IQR]	5 [3–6]	5 [3–6]	4 [3–6]	0.269
Anaemia * preoperatively (n, %)	88 (31.4)	39 (38.6)	49 (27.4)	0.061
Smoking (n, %)	137 (48.9)	46 (45.5)	91 (50.8)	0.455
CKD *—(n, %)	43 (15.4)	14 (13.9)	29 (16.2)	0.730
Diabetes (n, %)	80 (28.6)	29 (28.7)	51 (28.5)	1.000
Creatinine preoperatively (mg/dl)—median [IQR]	0.96 [0.79–1.15]	0.96 [0.74–1.16]	0.95 [0.82–1.14]	0.413
CPB duration (hours)—median [IQR]	1.55 [1.27–1.96]	1.55 [1.33–1.90]	1.56 [1.26–1.98]	0.946
Aortic clamping duration (hours)—median [IQR]	1.1 [0.85–1.41]	1.08 [0.88–1.36]	1.1 [0.81–1.43]	0.886
BMI preoperatively—kg/m^2^ (mean ± SD)	27.8 ± 4.9	27.7 ± 4.8	27.9 ± 5	0.752
Vaccination (n, %)	173 (61.8)	64 (63.4)	109 (60.8)	0.703

n—number of patients; IQR—interquartile range; ASA—American Society of Anesthesiologists physical status classification system; NYHA—New York Heart Association functional classification; EuroSCORE—European System for Cardiac Operative Risk Evaluation; CKD—chronic kidney disease; CPB—cardiopulmonary bypass; BMI—body mass index. Definitions: * Anaemia is a Hb < 12 g/dL in women and <13 g/dL in men; CKD = estimated glomerular filtration rate < 60 mL/min/1.73 m^2^; Vaccination: ≥1 dose of any WHO-authorized COVID-19 vaccine before surgery.

**Table 2:** Postoperative Outcomes by Prior COVID-19 Status.

Patients’ Outcomes	All Cohort—280 Patients	History of COVID-19—101 Patients	No History of COVID-19—179 Patients	*p*-Value
Vasopressor use * (n, %)	151 (53.9)	54 (53.5)	97 (54.2)	1.000
Inotrope use * (n, %)	81 (28.9)	31 (30.7)	50 (27.9)	0.681
Delirium * (n, %)	18 (6.4)	6 (5.9)	12 (6.7)	1.000
Stroke * (n, %)	3 (1.1)	0 (0)	3 (1.7)	0.555
Acute respiratory failure * (n, %)	65 (23.2)	20 (19.8)	45 (25.1)	0.377
Duration of invasive ventilation (hours)-median [IQR]	7 [5–10]	8 [5–12]	7 [5–10]	0.351
Pneumonia * (n, %)	9 (3.2)	3 (3)	6 (3.4)	1.000
Acute cardiac failure * (n, %)	30 (10.7)	11 (10.9)	19 (10.6)	1.000
Arrhythmias * (n, %)	65 (23.2)	25 (24.8)	40 (22.3)	0.661
AKI *—(n, %)	29 (10.4)	8 (7.9)	21 (11.7)	0.415
Hospitalization LOS—median [IQR]	8 [7–10]	8 [7–9]	8 [7–10]	0.132
ICU LOS—median [IQR]	3 [2–3]	3 [2–3]	3 [2–3]	0.919
Mortality (n, %)	5 (1.8)	1 (1)	4 (2.2)	0.657

n—number of patients; IQR—interquartile range; AKI—acute kidney injury; LOS—length of stay; Definitions: * vasopressor use: as any continuous infusion of norepinephrine, epinephrine, vasopressin, or equivalent maintained within the first 24 h of ICU admission; inotrope use: dobutamine or milrinone continuous infusion within the first 24 h of ICU admission; delirium: CAM-ICU positive; stroke: new focal deficit > 24 h with imaging confirmation; acute respiratory failure: re-intubation or non-invasive ventilation with PaO_2_/FiO_2_ < 300 mmHg; pneumonia: CDC/NHSN criteria; acute cardiac failure: low-output state requiring inotrope or mechanical support; arrhythmias: new atrial fibrillation/flutter or sustained ventricular arrhythmia requiring treatment; AKI: KDIGO serum creatinine criteria.

**Table 3:** The univariate and multivariate regression analysis for vasopressor use in the postoperative period.

Variables	Univariate		Multivariate	
	OR (95% CI)	*p*-Value	OR (95% CI)	*p*-Value
History of COVID-19 (yes)	0.971 (0.595, 1.584)	0.907	0.938 (0.556, 1.585)	0.812
CPB duration (hours)	2.589 (1.640, 4.089)	<0.001	2.797 (1.724, 4.535)	<0.001
Age (years)	1.023 (1.001, 1.045)	0.039	1.028 (1.004, 1.052)	0.02
Anaemia * (yes)	1.268 (0.762, 2.110)	0.361	1.193 (0.683, 2.084)	0.535
Obesity * (yes)	0.509 (0.303, 0.856)	0.011	0.496 (0.287, 0.598)	0.012
CKD * (yes)	1.224 (0.634, 2.361)	0.548	1.170 (0,566, 2.420)	0.672

Definitions: * Obesity is BMI > 30 kg/m^2^; Anaemia is a Hb < 12 g/dL in women and <13 g/dL in men; CKD = estimated glomerular filtration rate < 60 mL/min/1.73 m^2^.

## Data Availability

The data are not publicly available due to ethical restrictions involving patient confidentiality, but are available from the corresponding author upon reasonable request.

## Abbreviations

The following abbreviations are used in this manuscript:

CABG	Coronary Artery Bypass Graft
ICU	Intensive Care Unit
PASC	Post-Acute Sequelae of COVID-19
SD	Standard Deviation
